# Chromosome-level genome assembly of the Colorado potato beetle, *Leptinotarsa decemlineata*

**DOI:** 10.1038/s41597-023-01950-5

**Published:** 2023-01-19

**Authors:** Junjie Yan, Chaowei Zhang, Mengdi Zhang, Hang Zhou, Zhangqi Zuo, Xinhua Ding, Runzhi Zhang, Fei Li, Yulin Gao

**Affiliations:** 1 State Key Laboratory for Biology of Plant Diseases and Insect Pests, Institute of Plant Protection, Chinese Academy of Agricultural Sciences, Beijing, 100193 China; 2 State Key Laboratory of Rice Biology & Ministry of Agricultural and Rural Affairs Key Laboratory of Molecular Biology of Crop Pathogens and Insects & Key Laboratory of Biology of Crop Pathogens and Insects of Zhejiang Province, Institute of Insect Sciences, Zhejiang University, Hangzhou, 310058 China; 3 Institute of Plant Protection, Xinjiang Academy of Agricultural Sciences, Urumqi, 830091 China; 4 Key Laboratory of Zoological Systematics and Evolution, Institute of Zoology, Chinese Academy of Sciences, Beijing, 100101 China

**Keywords:** Genome, Agricultural genetics

## Abstract

The Colorado potato beetle (*Leptinotarsa decemlineata*) is one of the most notorious insect pests of potatoes globally. Here, we generated a high-quality chromosome-level genome assembly of *L. decemlineata* using a combination of the PacBio HiFi sequencing and Hi-C scaffolding technologies. The genome assembly (−1,008 Mb) is anchored to 18 chromosomes (17 + XO), with a scaffold N50 of 58.32 Mb. It contains 676 Mb repeat sequences and 29,606 protein-coding genes. The chromosome-level genome assembly of *L. decemlineata* provides in-depth knowledge and will be a helpful resource for the beetle and invasive biology research communities.

## Background & Summary

The Colorado potato beetle (CPB), *Leptinotarsa decemlineata*, is one of the most successful globally-invasive insects. Its current habitat ranges over 16 million km^2^ across North America, Europe and Asia and continues to expand globally^1^. Both adults and larvae devour entire leaves. This makes CPB one of the most destructive insect pests. It has been estimated that a single larva can destroy approximately 40 cm^2^ of potato leaves over the stage^2,3^. Chemical pesticides have been used to control CPB since the 1860s^4^. However, high selection pressures have promoted the emergence of high level insecticide resistant CPB populations over the last decades^5,6^. Since the middle of the last century, the beetle has developed resistance to 52 different insecticides compounds.

Whole-genome sequencing is a fundamental tool to address important scientific issues in biological research, by providing a whole set of gene resources of a given species. The first genome assembly of *L. decemlineata* based on Illumina short reads was published in 2018^7^, followed by an improved version Ldec_2.0. These two versions of CPB genomes have provided useful gene resources for the beetle community^8,9^. However, due to the limitation of short reads in genome assembly, the quality of the CPB genome still need be improved.

To this end, we applied the PacBio HiFi sequencing and High-throughput chromosome conformation capture technologies (Hi-C), to generate a high-quality chromosome-level genome assembly of *L. decemlineata* (Fig. 2). This produced a new CPB genome with high quality at chromosome level, which has a total scaffold length of 1,008.42 Mb mapping to 18 chromosomes (17 + XO). Compared to the published version Ldec_2.0, the scaffold N50 increased from 139 Kb to 58.32 Mb. Benchmarking Universal Single-Copy Orthologs (BUSCO) analysis showed that gene coverage increased from 92.1% to 98.0% (Table 1). A total of 676 Mb repeat sequences representing 67.04% of whole genome were identified, much more than that found in Ldec_2.0, suggesting the new version of CPB genome is more complete. Among these repeat sequences, 72.47% were classified as known repeat elements (Table 2). In addition, protein-coding genes increased from 24,671 to 29,606, showing that a more complete set of genes were obtained. Most protein-coding genes identified in the previous version can be found in the new annotation. Functional categories were classified based on the Kyoto Encyclopedia of Genes and Genomes (KEGG) pathway and Gene ontology (GO) databases (Table 3).

**Fig. 1:**
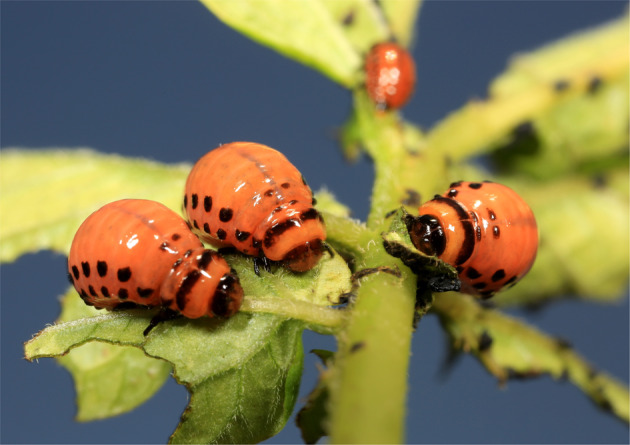
The Colorado potato beetle, *Leptinotarsa decemlineata*.

**Fig. 2:**
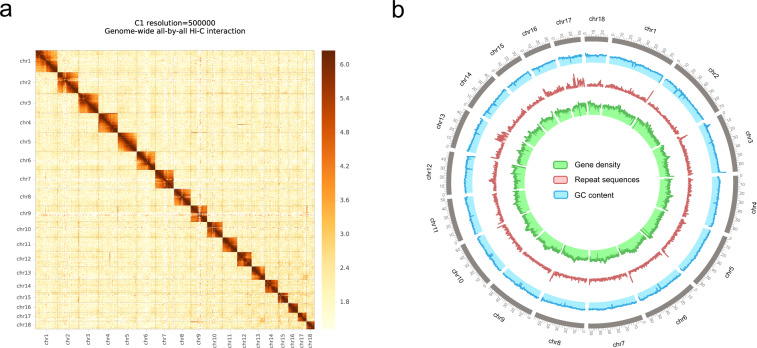
**Heatmap of genome-wide Hi-C data (resolution** = **500,000 bp) and overview of the genomic landscape of** ***Leptinotarsa decemlineata***. (**a**) The heatmap of chromosome interactions in *L. decemlineata* was visualized by HiCPlotter^45^. The frequency of Hi-C interaction links is represented by colours, which ranges from white (low) to red (high). (**b**) Circos plot of distribution of the genomic elements in *L. decemlineata* was visualized by Circos^46^. From the outer ring to the inner circle, blue, red and green represent GC content, repeat sequence coverage and gene density of each chromosome, respectively.

**Table 1:** Comparison of two *Leptinotarsa decemlineata* genome assemblies.

Genome assembly	This study	Ldec_2.0*
Genome size (Mb)	1008.42	641.99
Assembly level	Chromosome	Scaffold
Number of assembled chromosomes	18	Not available
Contig N50 (kb)	8098.89	47.4
Scaffold N50 (Mb)	58.32	0.139
Busco genes (%)	C:98.0% [S:89.5%, D:8.5%], F:0.8%, M:1.2%	C:92.1% [S:91.2%, D:0.9%], F:4.2%, M:3.7%
GC content (%)	34.7	22.3
Number of genes	29,606	24,671
Repeat (%)	67.04%	16.93%

*Ldec_2.0 is the previous published assembly from Sean^7^.

**Table 2:** Statistics of repeat elements of *Leptinotarsa decemlineata*.

Repeat types	Number of elements	Length occupied (bp)	Percentage of sequence
SINE	89	5392	0.00%
LINE	518279	175527570	17.41%
LTR	141193	66692839	6.61%
DNA elements	295376	156214793	15.49%
Unclassified	1110542	277582050	27.53%
Small RNA	512	38038	0.00%
Total base masked	2065991	676060682	67.04%

**Table 3:** Repeat elements of *Leptinotarsa decemlineata*.

Genome annotation	Number of elements
predicted protein-coding genes	29606
Swissprot	14053
GO	14606
KEGG	9135
Pfam	16628

A total of 418 single-copy orthologous genes were found among CPB and other 15 insect species (Table S1). These 1:1:1 orthologous gene were used to construct a phylogenetic tree. The evolutionary analysis results showed that *L. decemlineata* and other Chrysomelidae beetles formed a cluster. *Anoplophora glabripennis* (family: Cerambycidae) diverged from *L. decemlineata* (family: Chrysomelidae) approximately 96.5 million years ago (mya), and *Tribolium castaneum* (family: Tenebrionidae) diverged from *L. decemlineata* (family: Chrysomelidae) approximately 152.5 mya^9^.

**Fig. 3:**
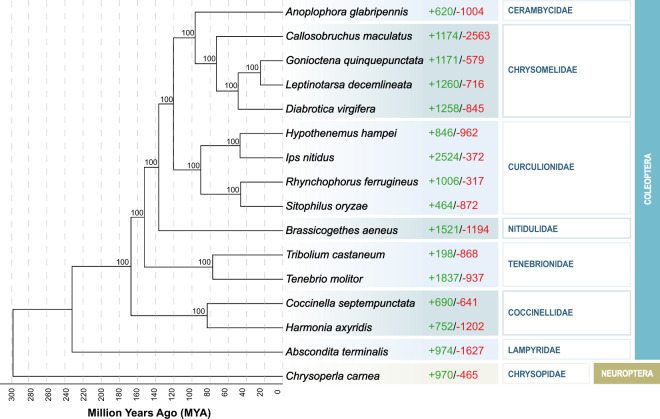
**Phylogenetic tree of** ***Leptinotarsa decemlineata*** **and 15 other insect species**. The numbers of expanded gene families (green) and contracted gene families (red) are shown to the right of each species branch^44^.

**Fig. 4:**
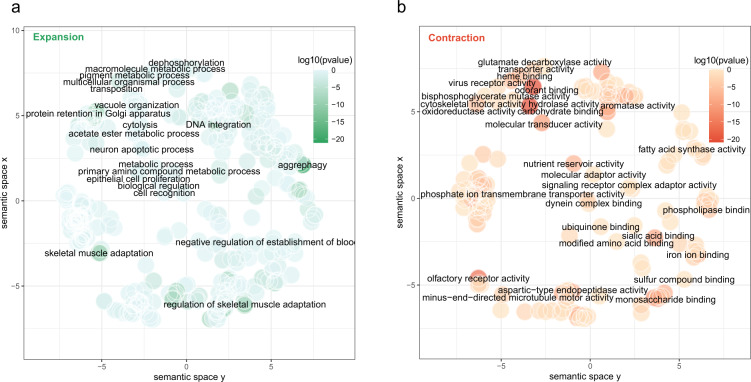
Gene ontology (GO) enrichment of expanded and contracted orthogroups in *Leptinotarsa decemlineata*.

In total, 14,446 gene clusters were identified across the 16 species. Compared with other insect species, CPB had 1,260 expanded and 716 contracted gene families (Fig. 3, Table S2). REVIGO analysis indicated that expanded orthogroups are enriched in DNA integration, macroautophagy, regulation of adenosine receptor signalling pathway and diverse biological process (Fig. 4a, Table S3). In contrast, the contracted orthogroups were significantly enriched in L-ornithine transmembrane, transporter activity, virus receptor activity (Fig. 4a, Table S4).

**Fig. 5:**
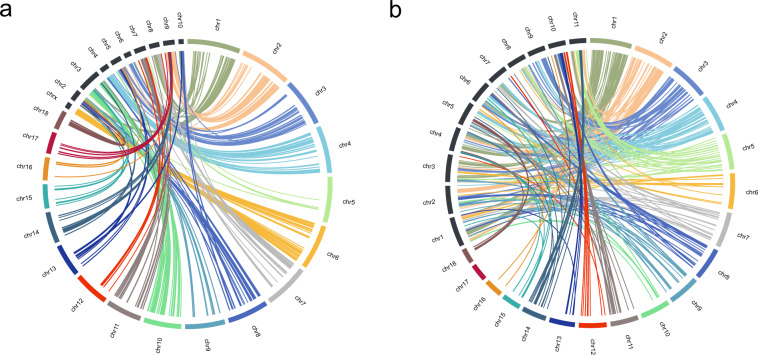
Comparative analysis of synteny among *Leptinotarsa decemlineata*, ***Tribolium castaneum*** and ***Anthonomus grandis***. (**a**) Whole-genome synteny between *Leptinotarsa decemlineata* and *Tribolium castaneum*. (**b**) Whole-genome synteny between *Leptinotarsa decemlineata* and *Anthonomus grandis*.

The whole genome of *Tribolium castaneum* and *Anthonomus grandis* in Chrysomelidae were publicly reported^10,11^, thus, we performed whole-genome synteny analysis of *L. decemlineata* with these two species. A large number of fission and fusion events were identified between *L. decemlineata* and the other two beetles, suggesting that the beetle family Chrysomelidae have undergone a high degree of divergence. CPB has XO sex determining system^12^. Synteny analysis also showed that the CPB Chromosome 6 (Chr 6) shared high sequence synteny with X chromosome of *T. castaneum* (Fig. 5). The gene LdVssc has been reported as X-linked^13^, and this gene can be found in Chr 6. Combining these evidences, the CPB Chr 6 is regarded as X chromosome.

As the first high-quality chromosome level genome assembly in Chrysomelidae, the chromosome-level genome assembly of *L. decemlineata* not only illuminate the genetic architecture of this important agricultural pests, providing a powerful approach to identify new gene targets for control measures, but also allows for exploration of biological characteristics of Chrysomelidae beetles.

## Methods

### Sample collection and sequencing

*Leptinotarsa decemlineata* adults were collected from Xinjiang Province, China. The adults were fed with fresh potato leaves and maintained at 26 ± 1 °C, under a 14:10-hr (light–dark) photoperiod cycle and 85% ± 5% relative humidity.

Genomic DNA was extracted from one female pupa using the QIAamp DNA Mini Kit (QIAGEN). Sex of the CPB pupa is identified by observing the 7th visible sternite^14^. The 7th visible sternite in the female pupa is separated in the middle by a suture, while the male pupa is complete and depressed in the centre. The integrity and purity of DNA was verified with agarose gel electrophoresis (AEG) and Nanodrop 2000. Eight micrograms of genomic DNA were sheared using g-Tubes (Covaris), and concentrated with AMPure PB magnetic beads. Each SMRT bell library was constructed using the Pacific Biosciences SMRT bell template prep kit 1.0. The constructed library was size-selected using the Sage ELF system for molecules 8–12 Kb, followed by primer annealing and the binding of SMRT bell templates to polymerases with the DNA Polymerase Binding Kit. Sequencing was carried out on the Pacific Bioscience Sequel II platform (Annoroad Gene Technology Co., Ltd, Beijing, China).

### Chromosome-level genome assembly of *L. decemlineata*

HiFi reads were produced using the circular consensus sequencing (CCS) mode on the PacBio long-read systems. 31 Gb HiFi reads (30×) were produced with an average length of 19,479 bp. *De novo* assembly of PacBio HiFi reads was performed using Hifiasm v0.13^14^.

Hi-C libraries were constructed and sequenced on the Illumina HiSeq X Ten platform (Annoroad Gene Technology Co., Ltd, Beijing, China), using a standard procedure^15^. The clean reads were first aligned to the genome assembly using bowtie 2 v2.2.3^16^. Unmapped reads were mainly composed of the chimeric regions spanning across the ligation junction. The ligation site of an unmapped read was determined with HiC-Pro v2.7.8^17^. Then, its 5′ fractions were aligned back with the genome assembly. A single alignment file which merged the results of both mapping steps was generated. Reads that had low mapping quality, multiple matches in the assembly, singletons and mitochondrial DNA were discarded. The valid interaction pairs were used to scaffold assembled contigs into 18 pseudo-chromosomes using LACHESIS v2e27abb^18^. The number of pseudochromosomes was consistent with the data of *L. decemlineata* karyotype (n = 17 + XO)^19^. The chromosome matrix was visualized as a heatmap in the form of diagonal patches of strong linkage (Fig. 2a). The quality and completeness of the assembled genome was evaluated using BUSCO v5.0^20^.

### Gene prediction and functional annotation

A repeat database was used to train RepeatModeler2^21^. Then, the repeat elements were annotated using the RepeatMasker v4.1.0^22^ by homology searching with default parameters. After filtering the repeat sequences, the results of *de novo* prediction, transcriptome-based and homolog-based methods were combined to predict gene composition^23^. *De novo* gene models were generated using BRAKER2 v.2.1.5^24^. Thirteen CPB transcriptomes were downloaded from the NCBI SRA database (SRR12121893, SRR13510813, SRR13510819, SRR13510821, SRR13510823, SRR9667707, SRR12121892, SRR13510812, SRR13510818, SRR13510820, SRR13510822, SRR9667699.1, SRR9667708). The transcriptomes were processed using Trimmomatic^25^, HISAT2 v.2.1.0^26^ and StringTie2 v.2.1.5^27^ to generate transcripts assemblies. The Homology proteins from all insect species were from OrthoDB^28^. Homology-based evidence was generated using GenomeThreader v.1.7.1^29^. Finally, gene models were predicted after integrating results of the three methods of predictions using EVidenceModeler^30^.

The functions of protein-coding genes were annotated using DIAMOND BLASTP against the Swiss-Prot protein database (https://www.uniprot.org/) and Pfam database (http://pfam.xfam.org/). The predicted genes were classified into functional categories based on KEGG (https://www.genome.jp/kegg) and GO (https://www.uniprot.org/) (Table 3).

### Phylogenetic analysis

We selected 15 coleopteran species for phylogenomic analysis, with *Chrysoperla carnea* (Order: Neuroptera) as an out-group. The protein sequences except CPB of these taxa were downloaded from NCBI and InsectBase 2.0^23^ (Table S1).

A total of 418 single-copy orthogroups were extracted using Broccoli v1.2^31^.The protein sequences in each orthogroup were extracted using seqkit v2.2.0^32^, independently aligned using MAFFT v7.471^33^ and filtered using trimAl v1.4^34^ with default parameters. The phylogenetic tree was constructed using iq-tree v1.6.10^35^ with the following parameters: -nt AUTO -m TEST -bb 1000. Branch support values were obtained from 1,000 bootstrap replicates. The divergence time among different species was estimated using the MCMCtree in the PAML package v4.9j^36^. Three standard divergence time points based on fossil records in the Paleobiology Database (www.paleobiodb.org) were applied: (a) stem Chrysomeloidea at 93.5–99.6 mya (b) stem Coleoptera at 166.1–168.3 mya (c) stem Coccinellidae at 295.5–298.9 mya.

### Gene family expansion and contraction

The expansion and contraction of gene families were determined using CAFE v5.0.029^37^. The results from the phylogenetic tree with divergence times were used as inputs. A p-value of 0.05 was used to identify families that were significantly expanded and contracted. Gene ontology (GO) enrichment of expanded and contracted orthogroups of *L.decemlineata* were analysed and visualized by REVIGO^38^. The dispensability (i.e., redundancy with respect to the chosen representative GO term) of GO terms was less than 0.1.

### Chromosomal synteny analysis

The whole-genome synteny analysis among the three species, was carried out using satsuma2 (https://github.com/bioinfologics/satsuma2). Synteny blocks were plotted across chromosomes using CIRCOS^39^.

### Identification of sex chromosomes

To determine X chromosome, Blastn was used to map the X-linked locus LdVssc with 18 CPB chromosomes with default parameters.

## Data Records

The PacBio and Hi-C sequencing data that were used for the genome assembly have been deposited in the NCBI Sequence Read Archive with accession number SRR20519124^40,41^ and SRR21095536^42^ and under BioProject accession number PRJNA854273. The chromosomal assembly has been deposited at GenBank with accession nember JANJPO000000000^43^. The annotated genes have been deposited in InsectBase 2.0 with ID IBG_00818^44^.

## Technical Validation

The chromosome-level genome assembly was 1,008 Mb with a scaffold N50 of 58.32 Mb. For quantitative assessment of genome assembly, BUSCO assessment showed that 98.0% of BUSCO genes (insecta_odb10) were successfully identified in the genome assembly (Table 1), suggesting a remarkably complete assembly of the *L. decemlineata* genome.

The Hi-C heatmap revealed a well-organized interaction contact pattern along the diagonals within/around the chromosome inversion region (Fig. 1), which indirectly confirmed the accuracy of the chromosome assembly.

## Supplementary information

Supplementary Information

## Data Availability

All software and pipelines were executed according to the manual and protocols of the published bioinformatic tools. The version and code/parameters of software have been described in Methods.
