# Supplementary material for: Chromosome-level genome assembly of the Colorado potato beetle, Leptinotarsa decemlineata
Source: Sci Data. 2023 Jan 19;10:36. doi: 10.1038/s41597-023-01950-5 (PMC9849343; doi:10.1038/s41597-023-01950-5)
Supplement: Supplementary file 1 — Supplementary Information [file 41597_2023_1950_MOESM1_ESM.docx]

# Table S1 Download links for protrein profiles used in phylogenomic analysis

| Species | Database | URL |
| --- | --- | --- |
| Abscondita terminalis | InsectBase 2.0 | <http://v2.insect-genome.com/Organism/2> |
| Anoplophora glabripennis | InsectBase 2.0 | <http://v2.insect-genome.com/Organism/63> |
| Brassicogethes aeneus | NCBI | <https://www.ncbi.nlm.nih.gov/data-hub/taxonomy/1431903/> |
| Chrysoperla carnea | InsectBase 2.0 | <http://v2.insect-genome.com/Organism/182> |
| Coccinella septempunctata | InsectBase 2.0 | <http://v2.insect-genome.com/Organism/194> |
| Diabrotica virgifera | InsectBase 2.0 | <http://v2.insect-genome.com/Organism/236> |
| Gonioctena quinquepunctata | InsectBase 2.0 | <http://v2.insect-genome.com/Organism/410> |
| Harmonia axyridis | InsectBase 2.0 | <http://v2.insect-genome.com/Organism/418> |
| Hypothenemus hampei | InsectBase 2.0 | <http://v2.insect-genome.com/Organism/465> |
| Ips nitidus | InsectBase 2.0 | <http://v2.insect-genome.com/Organism/468> |
| Rhynchophorus ferrugineus | InsectBase 2.0 | <http://v2.insect-genome.com/Organism/688> |
| Sitophilus oryzae | InsectBase 2.0 | <http://v2.insect-genome.com/Organism/708> |
| Tenebrio molitor | InsectBase 2.0 | <http://v2.insect-genome.com/Organism/732> |
| Tribolium castaneum | InsectBase 2.0 | <http://v2.insect-genome.com/Organism/768> |

# Table S2 Expanded and Contracted genes for each taxon

| #Taxon_ID | Expanded gens | Contracted genes |
| --- | --- | --- |
| <25> | 21 | 89 |
| <23> | 51 | 109 |
| Ldec<0> | 1260 | 716 |
| <12> | 181 | 95 |
| <29> | 23 | 159 |
| <27> | 64 | 224 |
| <15> | 111 | 258 |
| Cmac<9> | 1174 | 2563 |
| Init<6> | 2524 | 372 |
| Sory<4> | 464 | 872 |
| Agla<13> | 620 | 1004 |
| Csep<21> | 690 | 641 |
| Haxy<20> | 752 | 1202 |
| Tmol<16> | 1837 | 937 |
| Dvir<2> | 1258 | 845 |
| <10> | 116 | 414 |
| <19> | 65 | 62 |
| <3> | 436 | 212 |
| Gqui<1> | 1171 | 579 |
| Ater<26> | 974 | 1627 |
| Ccar<28> | 970 | 465 |
| Baen<18> | 1521 | 1194 |
| <22> | 304 | 582 |
| <8> | 353 | 109 |
| Rfer<5> | 1006 | 317 |
| Tcas<17> | 198 | 868 |
| <24> | 394 | 1106 |
| <14> | 144 | 259 |
| <11> | 549 | 263 |
| Hham<7> | 846 | 962 |

# Table S3 GO enrichment of expanded genes in *L.decemlineata* (p <0.05, FDR adjust p as p.adjust)

| GO ID | Description | out | All | pvalue | p.adjust |
| --- | --- | --- | --- | --- | --- |
| GO:0035973 | aggrephagy | 26 | 35 | 2.22E-25 | 8.28E-22 |
| GO:0015074 | DNA integration | 51 | 236 | 1.41E-16 | 2.64E-13 |
| GO:0045053 | protein retention in Golgi apparatus | 13 | 16 | 3.86E-14 | 4.80E-11 |
| GO:0016236 | macroautophagy | 37 | 164 | 5.83E-13 | 5.44E-10 |
| GO:0014733 | regulation of skeletal muscle adaptation | 11 | 13 | 1.72E-12 | 1.07E-09 |
| GO:0014883 | transition between fast and slow fiber | 11 | 13 | 1.72E-12 | 1.07E-09 |
| GO:0060167 | regulation of adenosine receptor signaling pathway | 9 | 9 | 7.44E-12 | 3.16E-09 |
| GO:0060168 | positive regulation of adenosine receptor signaling pathway | 9 | 9 | 7.44E-12 | 3.16E-09 |
| GO:0043501 | skeletal muscle adaptation | 11 | 14 | 7.63E-12 | 3.16E-09 |
| GO:0001973 | adenosine receptor signaling pathway | 9 | 10 | 7.05E-11 | 2.19E-08 |
| GO:0035588 | G-protein coupled purinergic receptor signaling pathway | 9 | 10 | 7.05E-11 | 2.19E-08 |
| GO:0045745 | positive regulation of G-protein coupled receptor protein signaling pathway | 9 | 10 | 7.05E-11 | 2.19E-08 |
| GO:0034067 | protein localization to Golgi apparatus | 14 | 30 | 2.81E-10 | 8.09E-08 |
| GO:0035587 | purinergic receptor signaling pathway | 9 | 11 | 3.68E-10 | 9.81E-08 |
| GO:0050679 | positive regulation of epithelial cell proliferation | 15 | 38 | 1.16E-09 | 2.90E-07 |
| GO:0046085 | adenosine metabolic process | 10 | 16 | 2.49E-09 | 5.28E-07 |
| GO:0071108 | protein K48-linked deubiquitination | 10 | 16 | 2.49E-09 | 5.28E-07 |
| GO:0014888 | striated muscle adaptation | 11 | 20 | 2.55E-09 | 5.28E-07 |
| GO:0006772 | thiamine metabolic process | 9 | 15 | 2.71E-08 | 5.32E-06 |
| GO:0006914 | autophagy | 40 | 271 | 4.77E-08 | 8.91E-06 |
| GO:0042723 | thiamine-containing compound metabolic process | 9 | 16 | 5.86E-08 | 9.52E-06 |
| GO:0051930 | regulation of sensory perception of pain | 9 | 16 | 5.86E-08 | 9.52E-06 |
| GO:0051931 | regulation of sensory perception | 9 | 16 | 5.86E-08 | 9.52E-06 |
| GO:0006313 | transposition, DNA-mediated | 29 | 165 | 7.93E-08 | 1.23E-05 |
| GO:0044057 | regulation of system process | 29 | 168 | 1.19E-07 | 1.78E-05 |
| GO:0043502 | regulation of muscle adaptation | 11 | 27 | 1.36E-07 | 1.95E-05 |
| GO:0070536 | protein K63-linked deubiquitination | 10 | 22 | 1.46E-07 | 2.01E-05 |
| GO:0001867 | complement activation, lectin pathway | 6 | 7 | 2.57E-07 | 3.42E-05 |
| GO:0006623 | protein targeting to vacuole | 13 | 41 | 3.11E-07 | 3.87E-05 |
| GO:0072666 | establishment of protein localization to vacuole | 13 | 41 | 3.11E-07 | 3.87E-05 |
| GO:0043500 | muscle adaptation | 11 | 29 | 3.23E-07 | 3.89E-05 |
| GO:0072665 | protein localization to vacuole | 13 | 46 | 1.37E-06 | 0.000159917 |
| GO:0006956 | complement activation | 6 | 9 | 2.78E-06 | 0.000305732 |
| GO:0072376 | protein activation cascade | 6 | 9 | 2.78E-06 | 0.000305732 |
| GO:0015766 | disaccharide transport | 19 | 98 | 3.08E-06 | 0.000311393 |
| GO:0015771 | trehalose transport | 19 | 98 | 3.08E-06 | 0.000311393 |
| GO:0015772 | oligosaccharide transport | 19 | 98 | 3.08E-06 | 0.000311393 |
| GO:0006378 | mRNA polyadenylation | 15 | 66 | 4.36E-06 | 0.000428868 |
| GO:0043631 | RNA polyadenylation | 15 | 67 | 5.32E-06 | 0.000509849 |
| GO:0032507 | maintenance of protein location in cell | 13 | 52 | 6.18E-06 | 0.000576812 |
| GO:0043170 | macromolecule metabolic process | 399 | 5907 | 8.77E-06 | 0.000798488 |
| GO:0006751 | glutathione catabolic process | 7 | 15 | 9.50E-06 | 0.000800387 |
| GO:0016063 | rhodopsin biosynthetic process | 7 | 15 | 9.50E-06 | 0.000800387 |
| GO:0046154 | rhodopsin metabolic process | 7 | 15 | 9.50E-06 | 0.000800387 |
| GO:0032196 | transposition | 29 | 207 | 9.64E-06 | 0.000800387 |
| GO:0019233 | sensory perception of pain | 9 | 27 | 1.34E-05 | 0.001090179 |
| GO:0006369 | termination of RNA polymerase II transcription | 6 | 11 | 1.38E-05 | 0.001099202 |
| GO:0042135 | neurotransmitter catabolic process | 7 | 16 | 1.60E-05 | 0.001230953 |
| GO:0006766 | vitamin metabolic process | 15 | 73 | 1.61E-05 | 0.001230953 |
| GO:0050678 | regulation of epithelial cell proliferation | 15 | 77 | 3.15E-05 | 0.002310255 |
| GO:0002229 | defense response to oomycetes | 5 | 8 | 3.22E-05 | 0.002310255 |
| GO:0002239 | response to oomycetes | 5 | 8 | 3.22E-05 | 0.002310255 |
| GO:0031644 | regulation of neurological system process | 10 | 38 | 4.54E-05 | 0.003199878 |
| GO:0045185 | maintenance of protein location | 13 | 62 | 4.71E-05 | 0.003256846 |
| GO:0051651 | maintenance of location in cell | 13 | 63 | 5.62E-05 | 0.003817491 |
| GO:0009597 | detection of virus | 5 | 9 | 6.89E-05 | 0.004218135 |
| GO:0030186 | melatonin metabolic process | 5 | 9 | 6.89E-05 | 0.004218135 |
| GO:0030187 | melatonin biosynthetic process | 5 | 9 | 6.89E-05 | 0.004218135 |
| GO:0042429 | serotonin catabolic process | 5 | 9 | 6.89E-05 | 0.004218135 |
| GO:0046334 | octopamine catabolic process | 5 | 9 | 6.89E-05 | 0.004218135 |
| GO:1901161 | primary amino compound catabolic process | 5 | 9 | 6.89E-05 | 0.004218135 |
| GO:0090257 | regulation of muscle system process | 13 | 65 | 7.92E-05 | 0.004769263 |
| GO:0019614 | catechol-containing compound catabolic process | 6 | 15 | 0.000122439 | 0.007035535 |
| GO:0042420 | dopamine catabolic process | 6 | 15 | 0.000122439 | 0.007035535 |
| GO:0042424 | catecholamine catabolic process | 6 | 15 | 0.000122439 | 0.007035535 |
| GO:0000393 | spliceosomal conformational changes to generate catalytic conformation | 5 | 10 | 0.000131167 | 0.007422875 |
| GO:0042133 | neurotransmitter metabolic process | 8 | 28 | 0.000140773 | 0.007847552 |
| GO:0008277 | regulation of G-protein coupled receptor protein signaling pathway | 10 | 44 | 0.000173443 | 0.009526629 |
| GO:0019336 | phenol-containing compound catabolic process | 6 | 16 | 0.000186282 | 0.0100835 |
| GO:0046333 | octopamine metabolic process | 6 | 17 | 0.000273767 | 0.014607429 |
| GO:0043171 | peptide catabolic process | 8 | 31 | 0.000305796 | 0.016023995 |
| GO:0040014 | regulation of multicellular organism growth | 10 | 47 | 0.000308896 | 0.016023995 |
| GO:0042219 | cellular modified amino acid catabolic process | 7 | 24 | 0.000321763 | 0.016462815 |
| GO:0007040 | lysosome organization | 10 | 48 | 0.000370125 | 0.018365127 |
| GO:0080171 | lytic vacuole organization | 10 | 48 | 0.000370125 | 0.018365127 |
| GO:0042436 | indole-containing compound catabolic process | 5 | 12 | 0.000373695 | 0.018365127 |
| GO:0032104 | regulation of response to extracellular stimulus | 14 | 87 | 0.000473394 | 0.022655207 |
| GO:0032107 | regulation of response to nutrient levels | 14 | 87 | 0.000473394 | 0.022655207 |
| GO:0042440 | pigment metabolic process | 18 | 129 | 0.000484121 | 0.022655207 |
| GO:0048069 | eye pigmentation | 8 | 33 | 0.000485252 | 0.022655207 |
| GO:0006726 | eye pigment biosynthetic process | 7 | 26 | 0.000552143 | 0.024550644 |
| GO:0042441 | eye pigment metabolic process | 7 | 26 | 0.000552143 | 0.024550644 |
| GO:0043324 | pigment metabolic process involved in developmental pigmentation | 7 | 26 | 0.000552143 | 0.024550644 |
| GO:0043474 | pigment metabolic process involved in pigmentation | 7 | 26 | 0.000552143 | 0.024550644 |
| GO:0016201 | synaptic target inhibition | 5 | 13 | 0.000578232 | 0.025063345 |
| GO:0035208 | positive regulation of hemocyte proliferation | 5 | 13 | 0.000578232 | 0.025063345 |
| GO:0002253 | activation of immune response | 15 | 99 | 0.000583805 | 0.025063345 |
| GO:0072527 | pyrimidine-containing compound metabolic process | 13 | 79 | 0.000599592 | 0.025448581 |
| GO:0032496 | response to lipopolysaccharide | 16 | 110 | 0.000617 | 0.025617647 |
| GO:0006767 | water-soluble vitamin metabolic process | 10 | 51 | 0.000617293 | 0.025617647 |
| GO:0007033 | vacuole organization | 17 | 121 | 0.000635118 | 0.026067746 |
| GO:0051239 | regulation of multicellular organismal process | 98 | 1226 | 0.000646972 | 0.026086704 |
| GO:0044260 | cellular macromolecule metabolic process | 363 | 5520 | 0.000649548 | 0.026086704 |
| GO:0021516 | dorsal spinal cord development | 4 | 8 | 0.000665064 | 0.026147498 |
| GO:0043654 | recognition of apoptotic cell | 4 | 8 | 0.000665064 | 0.026147498 |
| GO:0050673 | epithelial cell proliferation | 15 | 101 | 0.00072404 | 0.02796684 |
| GO:0021527 | spinal cord association neuron differentiation | 3 | 4 | 0.000756265 | 0.02796684 |
| GO:0031179 | peptide modification | 3 | 4 | 0.000756265 | 0.02796684 |
| GO:0044179 | hemolysis in other organism | 3 | 4 | 0.000756265 | 0.02796684 |
| GO:0051715 | cytolysis in other organism | 3 | 4 | 0.000756265 | 0.02796684 |
| GO:0061017 | hepatoblast differentiation | 3 | 4 | 0.000756265 | 0.02796684 |
| GO:0040015 | negative regulation of multicellular organism growth | 5 | 14 | 0.000856548 | 0.031060275 |
| GO:2001244 | positive regulation of intrinsic apoptotic signaling pathway | 5 | 14 | 0.000856548 | 0.031060275 |
| GO:0002237 | response to molecule of bacterial origin | 16 | 114 | 0.00091779 | 0.032961009 |
| GO:0032365 | intracellular lipid transport | 7 | 29 | 0.001124351 | 0.039617477 |
| GO:0044273 | sulfur compound catabolic process | 7 | 29 | 0.001124351 | 0.039617477 |
| GO:0030212 | hyaluronan metabolic process | 4 | 9 | 0.001142002 | 0.039863338 |
| GO:0006379 | mRNA cleavage | 6 | 22 | 0.001284632 | 0.043619082 |
| GO:0032366 | intracellular sterol transport | 6 | 22 | 0.001284632 | 0.043619082 |
| GO:0032367 | intracellular cholesterol transport | 6 | 22 | 0.001284632 | 0.043619082 |
| GO:0046148 | pigment biosynthetic process | 14 | 98 | 0.001580868 | 0.053194086 |
| GO:0042417 | dopamine metabolic process | 6 | 23 | 0.001653368 | 0.055136869 |
| GO:0042428 | serotonin metabolic process | 5 | 16 | 0.001695113 | 0.055537251 |
| GO:0046958 | nonassociative learning | 5 | 16 | 0.001695113 | 0.055537251 |
| GO:0000350 | generation of catalytic spliceosome for second transesterification step | 3 | 5 | 0.001808536 | 0.056519629 |
| GO:0021854 | hypothalamus development | 3 | 5 | 0.001808536 | 0.056519629 |
| GO:1901748 | leukotriene D4 metabolic process | 3 | 5 | 0.001808536 | 0.056519629 |
| GO:1901750 | leukotriene D4 biosynthetic process | 3 | 5 | 0.001808536 | 0.056519629 |
| GO:0006910 | phagocytosis, recognition | 4 | 10 | 0.001815892 | 0.056519629 |
| GO:0008228 | opsonization | 4 | 10 | 0.001815892 | 0.056519629 |
| GO:0050830 | defense response to Gram-positive bacterium | 8 | 40 | 0.001873053 | 0.057816959 |
| GO:0008284 | positive regulation of cell proliferation | 29 | 281 | 0.001986535 | 0.060817295 |
| GO:0042745 | circadian sleep/wake cycle | 9 | 50 | 0.002152521 | 0.065363147 |
| GO:0002221 | pattern recognition receptor signaling pathway | 8 | 41 | 0.002210485 | 0.066581954 |
| GO:1901160 | primary amino compound metabolic process | 5 | 17 | 0.00228734 | 0.068345729 |
| GO:0007034 | vacuolar transport | 14 | 102 | 0.00232494 | 0.068917876 |
| GO:0006750 | glutathione biosynthetic process | 4 | 11 | 0.002722717 | 0.076461275 |
| GO:0019184 | nonribosomal peptide biosynthetic process | 4 | 11 | 0.002722717 | 0.076461275 |
| GO:0042360 | vitamin E metabolic process | 4 | 11 | 0.002722717 | 0.076461275 |
| GO:0046959 | habituation | 4 | 11 | 0.002722717 | 0.076461275 |
| GO:0090212 | negative regulation of establishment of blood-brain barrier | 4 | 11 | 0.002722717 | 0.076461275 |
| GO:1900221 | regulation of beta-amyloid clearance | 4 | 11 | 0.002722717 | 0.076461275 |
| GO:1900223 | positive regulation of beta-amyloid clearance | 4 | 11 | 0.002722717 | 0.076461275 |
| GO:0051235 | maintenance of location | 18 | 151 | 0.003010407 | 0.081652599 |
| GO:0002347 | response to tumor cell | 5 | 18 | 0.003016883 | 0.081652599 |
| GO:0007593 | chitin-based cuticle sclerotization | 5 | 18 | 0.003016883 | 0.081652599 |
| GO:0042402 | cellular biogenic amine catabolic process | 5 | 18 | 0.003016883 | 0.081652599 |
| GO:0042435 | indole-containing compound biosynthetic process | 5 | 18 | 0.003016883 | 0.081652599 |
| GO:0006959 | humoral immune response | 13 | 94 | 0.003070093 | 0.082494955 |
| GO:0046901 | tetrahydrofolylpolyglutamate biosynthetic process | 2 | 2 | 0.003398191 | 0.085182849 |
| GO:0048251 | elastic fiber assembly | 2 | 2 | 0.003398191 | 0.085182849 |
| GO:0070669 | response to interleukin-2 | 2 | 2 | 0.003398191 | 0.085182849 |
| GO:0071352 | cellular response to interleukin-2 | 2 | 2 | 0.003398191 | 0.085182849 |
| GO:0080148 | negative regulation of response to water deprivation | 2 | 2 | 0.003398191 | 0.085182849 |
| GO:1904567 | response to wortmannin | 2 | 2 | 0.003398191 | 0.085182849 |
| GO:1904568 | cellular response to wortmannin | 2 | 2 | 0.003398191 | 0.085182849 |
| GO:1990637 | response to prolactin | 2 | 2 | 0.003398191 | 0.085182849 |
| GO:1990646 | cellular response to prolactin | 2 | 2 | 0.003398191 | 0.085182849 |
| GO:2000070 | regulation of response to water deprivation | 2 | 2 | 0.003398191 | 0.085182849 |
| GO:0019370 | leukotriene biosynthetic process | 3 | 6 | 0.003460425 | 0.086164585 |
| GO:0032095 | regulation of response to food | 4 | 12 | 0.003897229 | 0.09521738 |
| GO:0035721 | intraciliary retrograde transport | 4 | 12 | 0.003897229 | 0.09521738 |
| GO:0009310 | amine catabolic process | 5 | 19 | 0.003900471 | 0.09521738 |
| GO:0070997 | neuron death | 14 | 108 | 0.003963795 | 0.096134899 |
| GO:0008643 | carbohydrate transport | 19 | 168 | 0.004226063 | 0.101834482 |
| GO:1901360 | organic cyclic compound metabolic process | 283 | 4291 | 0.004300204 | 0.102956804 |
| GO:0031124 | mRNA 3'-end processing | 15 | 121 | 0.004480204 | 0.106583199 |
| GO:0071704 | organic substance metabolic process | 472 | 7552 | 0.00457793 | 0.108218795 |
| GO:0002224 | toll-like receptor signaling pathway | 6 | 28 | 0.004811604 | 0.11022853 |
| GO:0007614 | short-term memory | 6 | 28 | 0.004811604 | 0.11022853 |
| GO:0051402 | neuron apoptotic process | 11 | 77 | 0.004852577 | 0.11022853 |
| GO:0002804 | positive regulation of antifungal peptide production | 5 | 20 | 0.004954771 | 0.11022853 |
| GO:0006779 | porphyrin-containing compound biosynthetic process | 5 | 20 | 0.004954771 | 0.11022853 |
| GO:0006783 | heme biosynthetic process | 5 | 20 | 0.004954771 | 0.11022853 |
| GO:0033014 | tetrapyrrole biosynthetic process | 5 | 20 | 0.004954771 | 0.11022853 |
| GO:1902875 | regulation of embryonic pattern specification | 5 | 20 | 0.004954771 | 0.11022853 |
| GO:2000785 | regulation of autophagosome assembly | 5 | 20 | 0.004954771 | 0.11022853 |
| GO:0046483 | heterocycle metabolic process | 269 | 4067 | 0.00495807 | 0.11022853 |
| GO:0042749 | regulation of circadian sleep/wake cycle | 8 | 47 | 0.005348656 | 0.115986615 |
| GO:0006241 | CTP biosynthetic process | 4 | 13 | 0.005372339 | 0.115986615 |
| GO:0021536 | diencephalon development | 4 | 13 | 0.005372339 | 0.115986615 |
| GO:0046036 | CTP metabolic process | 4 | 13 | 0.005372339 | 0.115986615 |
| GO:0097242 | beta-amyloid clearance | 4 | 13 | 0.005372339 | 0.115986615 |
| GO:0006353 | DNA-templated transcription, termination | 6 | 29 | 0.005773497 | 0.122269203 |
| GO:0044088 | regulation of vacuole organization | 6 | 29 | 0.005773497 | 0.122269203 |
| GO:0060086 | circadian temperature homeostasis | 3 | 7 | 0.005794284 | 0.122269203 |
| GO:0070365 | hepatocyte differentiation | 3 | 7 | 0.005794284 | 0.122269203 |
| GO:0002781 | antifungal peptide production | 5 | 21 | 0.006196206 | 0.124423809 |
| GO:0002788 | regulation of antifungal peptide production | 5 | 21 | 0.006196206 | 0.124423809 |
| GO:0006775 | fat-soluble vitamin metabolic process | 5 | 21 | 0.006196206 | 0.124423809 |
| GO:0006778 | porphyrin-containing compound metabolic process | 5 | 21 | 0.006196206 | 0.124423809 |
| GO:0007141 | male meiosis I | 5 | 21 | 0.006196206 | 0.124423809 |
| GO:0019732 | antifungal humoral response | 5 | 21 | 0.006196206 | 0.124423809 |
| GO:0031648 | protein destabilization | 5 | 21 | 0.006196206 | 0.124423809 |
| GO:0042168 | heme metabolic process | 5 | 21 | 0.006196206 | 0.124423809 |
| GO:1900150 | regulation of defense response to fungus | 5 | 21 | 0.006196206 | 0.124423809 |
| GO:0044237 | cellular metabolic process | 450 | 7188 | 0.00641651 | 0.128158632 |
| GO:0034641 | cellular nitrogen compound metabolic process | 308 | 4743 | 0.006620012 | 0.131519922 |
| GO:0003333 | amino acid transmembrane transport | 7 | 39 | 0.006678581 | 0.13198149 |
| GO:0048640 | negative regulation of developmental growth | 11 | 81 | 0.00713802 | 0.139647265 |
| GO:0006189 | 'de novo' IMP biosynthetic process | 4 | 14 | 0.007178655 | 0.139647265 |
| GO:0090210 | regulation of establishment of blood-brain barrier | 4 | 14 | 0.007178655 | 0.139647265 |
| GO:0003012 | muscle system process | 13 | 104 | 0.007328916 | 0.141831606 |
| GO:0006725 | cellular aromatic compound metabolic process | 272 | 4147 | 0.007492748 | 0.144254718 |
| GO:0035212 | cell competition in a multicellular organism | 5 | 22 | 0.007640786 | 0.146350447 |
| GO:0016241 | regulation of macroautophagy | 10 | 71 | 0.007830242 | 0.147965819 |
| GO:0007602 | phototransduction | 8 | 50 | 0.007835041 | 0.147965819 |
| GO:0050778 | positive regulation of immune response | 19 | 178 | 0.007843971 | 0.147965819 |
| GO:1903825 | organic acid transmembrane transport | 8 | 51 | 0.008831048 | 0.161947228 |
| GO:0000389 | mRNA 3'-splice site recognition | 3 | 8 | 0.008871815 | 0.161947228 |
| GO:0006188 | IMP biosynthetic process | 4 | 15 | 0.009344128 | 0.161947228 |
| GO:0035001 | dorsal trunk growth, open tracheal system | 4 | 15 | 0.009344128 | 0.161947228 |
| GO:0045542 | positive regulation of cholesterol biosynthetic process | 4 | 15 | 0.009344128 | 0.161947228 |
| GO:0048583 | regulation of response to stimulus | 116 | 1621 | 0.009600873 | 0.161947228 |
| GO:0007256 | activation of JNKK activity | 2 | 3 | 0.009799216 | 0.161947228 |
| GO:0015816 | glycine transport | 2 | 3 | 0.009799216 | 0.161947228 |
| GO:0019919 | peptidyl-arginine methylation, to asymmetrical-dimethyl arginine | 2 | 3 | 0.009799216 | 0.161947228 |
| GO:0021590 | cerebellum maturation | 2 | 3 | 0.009799216 | 0.161947228 |
| GO:0021691 | cerebellar Purkinje cell layer maturation | 2 | 3 | 0.009799216 | 0.161947228 |
| GO:0021699 | cerebellar cortex maturation | 2 | 3 | 0.009799216 | 0.161947228 |
| GO:0021942 | radial glia guided migration of Purkinje cell | 2 | 3 | 0.009799216 | 0.161947228 |
| GO:0021984 | adenohypophysis development | 2 | 3 | 0.009799216 | 0.161947228 |
| GO:0035080 | heat shock-mediated polytene chromosome puffing | 2 | 3 | 0.009799216 | 0.161947228 |
| GO:0035246 | peptidyl-arginine N-methylation | 2 | 3 | 0.009799216 | 0.161947228 |
| GO:0035247 | peptidyl-arginine omega-N-methylation | 2 | 3 | 0.009799216 | 0.161947228 |
| GO:0035675 | neuromast hair cell development | 2 | 3 | 0.009799216 | 0.161947228 |
| GO:0035677 | posterior lateral line neuromast hair cell development | 2 | 3 | 0.009799216 | 0.161947228 |
| GO:0046900 | tetrahydrofolylpolyglutamate metabolic process | 2 | 3 | 0.009799216 | 0.161947228 |
| GO:0048886 | neuromast hair cell differentiation | 2 | 3 | 0.009799216 | 0.161947228 |
| GO:0048923 | posterior lateral line neuromast hair cell differentiation | 2 | 3 | 0.009799216 | 0.161947228 |
| GO:0051450 | myoblast proliferation | 2 | 3 | 0.009799216 | 0.161947228 |
| GO:1904936 | interneuron migration | 2 | 3 | 0.009799216 | 0.161947228 |
| GO:1904937 | sensory neuron migration | 2 | 3 | 0.009799216 | 0.161947228 |
| GO:1904983 | transmembrane glycine transport from cytosol to mitochondrion | 2 | 3 | 0.009799216 | 0.161947228 |
| GO:2000288 | positive regulation of myoblast proliferation | 2 | 3 | 0.009799216 | 0.161947228 |
| GO:2000291 | regulation of myoblast proliferation | 2 | 3 | 0.009799216 | 0.161947228 |
| GO:0032501 | multicellular organismal process | 278 | 4269 | 0.009943207 | 0.163602985 |
| GO:0016579 | protein deubiquitination | 11 | 86 | 0.011087399 | 0.18108562 |
| GO:0002758 | innate immune response-activating signal transduction | 8 | 53 | 0.011102706 | 0.18108562 |
| GO:0009209 | pyrimidine ribonucleoside triphosphate biosynthetic process | 4 | 16 | 0.011893799 | 0.192308836 |
| GO:0046040 | IMP metabolic process | 4 | 16 | 0.011893799 | 0.192308836 |
| GO:0048584 | positive regulation of response to stimulus | 63 | 814 | 0.012112664 | 0.195003451 |
| GO:0017182 | peptidyl-diphthamide metabolic process | 3 | 9 | 0.012736722 | 0.199592055 |
| GO:0017183 | peptidyl-diphthamide biosynthetic process from peptidyl-histidine | 3 | 9 | 0.012736722 | 0.199592055 |
| GO:0019344 | cysteine biosynthetic process | 3 | 9 | 0.012736722 | 0.199592055 |
| GO:0019835 | cytolysis | 3 | 9 | 0.012736722 | 0.199592055 |
| GO:0002576 | platelet degranulation | 6 | 34 | 0.012771754 | 0.199592055 |
| GO:0006584 | catecholamine metabolic process | 6 | 34 | 0.012771754 | 0.199592055 |
| GO:0009712 | catechol-containing compound metabolic process | 6 | 34 | 0.012771754 | 0.199592055 |
| GO:0042430 | indole-containing compound metabolic process | 5 | 25 | 0.013344223 | 0.206807768 |
| GO:0050919 | negative chemotaxis | 5 | 25 | 0.013344223 | 0.206807768 |
| GO:0006259 | DNA metabolic process | 72 | 958 | 0.014459016 | 0.223158775 |
| GO:0009208 | pyrimidine ribonucleoside triphosphate metabolic process | 4 | 17 | 0.014849633 | 0.224525644 |
| GO:0030536 | larval feeding behavior | 4 | 17 | 0.014849633 | 0.224525644 |
| GO:0032094 | response to food | 4 | 17 | 0.014849633 | 0.224525644 |
| GO:0045540 | regulation of cholesterol biosynthetic process | 4 | 17 | 0.014849633 | 0.224525644 |
| GO:0090205 | positive regulation of cholesterol metabolic process | 4 | 17 | 0.014849633 | 0.224525644 |
| GO:0031123 | RNA 3'-end processing | 16 | 151 | 0.014908262 | 0.224525644 |
| GO:0006511 | ubiquitin-dependent protein catabolic process | 40 | 483 | 0.015580451 | 0.233706764 |
| GO:0045187 | regulation of circadian sleep/wake cycle, sleep | 5 | 26 | 0.015748225 | 0.234341114 |
| GO:1902930 | regulation of alcohol biosynthetic process | 5 | 26 | 0.015748225 | 0.234341114 |
| GO:0072594 | establishment of protein localization to organelle | 23 | 245 | 0.016360923 | 0.242492254 |
| GO:0008039 | synaptic target recognition | 6 | 36 | 0.01676659 | 0.246548085 |
| GO:0045676 | regulation of R7 cell differentiation | 6 | 36 | 0.01676659 | 0.246548085 |
| GO:0034374 | low-density lipoprotein particle remodeling | 3 | 10 | 0.017417078 | 0.253123686 |
| GO:0034436 | glycoprotein transport | 3 | 10 | 0.017417078 | 0.253123686 |
| GO:0055091 | phospholipid homeostasis | 3 | 10 | 0.017417078 | 0.253123686 |
| GO:0009744 | response to sucrose | 4 | 18 | 0.018230419 | 0.254063163 |
| GO:0010893 | positive regulation of steroid biosynthetic process | 4 | 18 | 0.018230419 | 0.254063163 |
| GO:0021515 | cell differentiation in spinal cord | 4 | 18 | 0.018230419 | 0.254063163 |
| GO:1902932 | positive regulation of alcohol biosynthetic process | 4 | 18 | 0.018230419 | 0.254063163 |
| GO:1901616 | organic hydroxy compound catabolic process | 9 | 69 | 0.018277989 | 0.254063163 |
| GO:0009617 | response to bacterium | 28 | 317 | 0.018544909 | 0.254063163 |
| GO:0000349 | generation of catalytic spliceosome for first transesterification step | 2 | 4 | 0.018842168 | 0.254063163 |
| GO:0006581 | acetylcholine catabolic process | 2 | 4 | 0.018842168 | 0.254063163 |
| GO:0021534 | cell proliferation in hindbrain | 2 | 4 | 0.018842168 | 0.254063163 |
| GO:0021924 | cell proliferation in external granule layer | 2 | 4 | 0.018842168 | 0.254063163 |
| GO:0021930 | cerebellar granule cell precursor proliferation | 2 | 4 | 0.018842168 | 0.254063163 |
| GO:0021932 | hindbrain radial glia guided cell migration | 2 | 4 | 0.018842168 | 0.254063163 |
| GO:0035079 | polytene chromosome puffing | 2 | 4 | 0.018842168 | 0.254063163 |
| GO:0044210 | 'de novo' CTP biosynthetic process | 2 | 4 | 0.018842168 | 0.254063163 |
| GO:0045200 | establishment of neuroblast polarity | 2 | 4 | 0.018842168 | 0.254063163 |
| GO:0048755 | branching morphogenesis of a nerve | 2 | 4 | 0.018842168 | 0.254063163 |
| GO:0090025 | regulation of monocyte chemotaxis | 2 | 4 | 0.018842168 | 0.254063163 |
| GO:0090026 | positive regulation of monocyte chemotaxis | 2 | 4 | 0.018842168 | 0.254063163 |
| GO:0097402 | neuroblast migration | 2 | 4 | 0.018842168 | 0.254063163 |
| GO:2000672 | negative regulation of motor neuron apoptotic process | 2 | 4 | 0.018842168 | 0.254063163 |
| GO:0009581 | detection of external stimulus | 10 | 81 | 0.018975082 | 0.254935003 |
| GO:0045089 | positive regulation of innate immune response | 11 | 93 | 0.01918261 | 0.256799455 |
| GO:0009583 | detection of light stimulus | 8 | 59 | 0.02049413 | 0.272800806 |
| GO:0009582 | detection of abiotic stimulus | 10 | 82 | 0.02052397 | 0.272800806 |
| GO:0045995 | regulation of embryonic development | 11 | 94 | 0.0206245 | 0.273164918 |
| GO:0007601 | visual perception | 13 | 119 | 0.021192861 | 0.27925987 |
| GO:0021510 | spinal cord development | 5 | 28 | 0.021383754 | 0.27925987 |
| GO:0033013 | tetrapyrrole metabolic process | 5 | 28 | 0.021383754 | 0.27925987 |
| GO:0050802 | circadian sleep/wake cycle, sleep | 5 | 28 | 0.021383754 | 0.27925987 |
| GO:0001892 | embryonic placenta development | 4 | 19 | 0.022051738 | 0.284011179 |
| GO:0034285 | response to disaccharide | 4 | 19 | 0.022051738 | 0.284011179 |
| GO:0072401 | signal transduction involved in DNA integrity checkpoint | 4 | 19 | 0.022051738 | 0.284011179 |
| GO:0072422 | signal transduction involved in DNA damage checkpoint | 4 | 19 | 0.022051738 | 0.284011179 |
| GO:0019941 | modification-dependent protein catabolic process | 40 | 495 | 0.022327594 | 0.286575826 |
| GO:0006807 | nitrogen compound metabolic process | 318 | 5014 | 0.022804951 | 0.287362673 |
| GO:0006782 | protoporphyrinogen IX biosynthetic process | 3 | 11 | 0.022927464 | 0.287362673 |
| GO:0010745 | negative regulation of macrophage derived foam cell differentiation | 3 | 11 | 0.022927464 | 0.287362673 |
| GO:0032536 | regulation of cell projection size | 3 | 11 | 0.022927464 | 0.287362673 |
| GO:0046501 | protoporphyrinogen IX metabolic process | 3 | 11 | 0.022927464 | 0.287362673 |
| GO:0097421 | liver regeneration | 3 | 11 | 0.022927464 | 0.287362673 |
| GO:1902004 | positive regulation of beta-amyloid formation | 3 | 11 | 0.022927464 | 0.287362673 |
| GO:0070646 | protein modification by small protein removal | 11 | 96 | 0.023745431 | 0.29661935 |
| GO:0007186 | G-protein coupled receptor signaling pathway | 24 | 268 | 0.024071883 | 0.29969494 |
| GO:0002218 | activation of innate immune response | 8 | 61 | 0.024603155 | 0.305291639 |
| GO:0007498 | mesoderm development | 9 | 73 | 0.025526458 | 0.315699734 |
| GO:0008152 | metabolic process | 483 | 7882 | 0.025639406 | 0.316035252 |
| GO:0048066 | developmental pigmentation | 10 | 85 | 0.025722816 | 0.316035252 |
| GO:0006605 | protein targeting | 24 | 270 | 0.026024346 | 0.316165708 |
| GO:0009148 | pyrimidine nucleoside triphosphate biosynthetic process | 4 | 20 | 0.02632598 | 0.316165708 |
| GO:0009268 | response to pH | 4 | 20 | 0.02632598 | 0.316165708 |
| GO:0031062 | positive regulation of histone methylation | 4 | 20 | 0.02632598 | 0.316165708 |
| GO:0035011 | melanotic encapsulation of foreign target | 4 | 20 | 0.02632598 | 0.316165708 |
| GO:0045940 | positive regulation of steroid metabolic process | 4 | 20 | 0.02632598 | 0.316165708 |
| GO:0072395 | signal transduction involved in cell cycle checkpoint | 4 | 20 | 0.02632598 | 0.316165708 |
| GO:0046164 | alcohol catabolic process | 8 | 62 | 0.02685984 | 0.321543278 |
| GO:0042398 | cellular modified amino acid biosynthetic process | 6 | 40 | 0.027171234 | 0.324231823 |
| GO:0007275 | multicellular organism development | 215 | 3300 | 0.027510895 | 0.327239462 |
| GO:0048512 | circadian behavior | 9 | 74 | 0.027617418 | 0.327463674 |
| GO:0043632 | modification-dependent macromolecule catabolic process | 40 | 503 | 0.027992092 | 0.330855899 |
| GO:0006534 | cysteine metabolic process | 3 | 12 | 0.029270875 | 0.338706424 |
| GO:0010743 | regulation of macrophage derived foam cell differentiation | 3 | 12 | 0.029270875 | 0.338706424 |
| GO:0010872 | regulation of cholesterol esterification | 3 | 12 | 0.029270875 | 0.338706424 |
| GO:0018202 | peptidyl-histidine modification | 3 | 12 | 0.029270875 | 0.338706424 |
| GO:0033700 | phospholipid efflux | 3 | 12 | 0.029270875 | 0.338706424 |
| GO:0006310 | DNA recombination | 33 | 402 | 0.029414012 | 0.338706424 |
| GO:0002076 | osteoblast development | 2 | 5 | 0.030197922 | 0.338706424 |
| GO:0006610 | ribosomal protein import into nucleus | 2 | 5 | 0.030197922 | 0.338706424 |
| GO:0007510 | cardioblast cell fate determination | 2 | 5 | 0.030197922 | 0.338706424 |
| GO:0021983 | pituitary gland development | 2 | 5 | 0.030197922 | 0.338706424 |
| GO:0035469 | determination of pancreatic left/right asymmetry | 2 | 5 | 0.030197922 | 0.338706424 |
| GO:0061026 | cardiac muscle tissue regeneration | 2 | 5 | 0.030197922 | 0.338706424 |
| GO:0071910 | determination of liver left/right asymmetry | 2 | 5 | 0.030197922 | 0.338706424 |
| GO:0090158 | endoplasmic reticulum membrane organization | 2 | 5 | 0.030197922 | 0.338706424 |
| GO:0090594 | inflammatory response to wounding | 2 | 5 | 0.030197922 | 0.338706424 |
| GO:0097475 | motor neuron migration | 2 | 5 | 0.030197922 | 0.338706424 |
| GO:2000671 | regulation of motor neuron apoptotic process | 2 | 5 | 0.030197922 | 0.338706424 |
| GO:0030301 | cholesterol transport | 6 | 41 | 0.030315839 | 0.339010959 |
| GO:0042752 | regulation of circadian rhythm | 11 | 100 | 0.030996957 | 0.342512876 |
| GO:0007588 | excretion | 4 | 21 | 0.031062397 | 0.342512876 |
| GO:0010888 | negative regulation of lipid storage | 4 | 21 | 0.031062397 | 0.342512876 |
| GO:0090181 | regulation of cholesterol metabolic process | 4 | 21 | 0.031062397 | 0.342512876 |
| GO:0048638 | regulation of developmental growth | 20 | 219 | 0.031087514 | 0.342512876 |
| GO:0032101 | regulation of response to external stimulus | 29 | 346 | 0.031207488 | 0.342823429 |
| GO:0050953 | sensory perception of light stimulus | 13 | 126 | 0.032089034 | 0.351473732 |
| GO:0006139 | nucleobase-containing compound metabolic process | 252 | 3932 | 0.032530406 | 0.355266272 |
| GO:0002684 | positive regulation of immune system process | 22 | 249 | 0.034003323 | 0.370269424 |
| GO:0031099 | regeneration | 8 | 65 | 0.034482468 | 0.374395403 |
| GO:0003008 | system process | 59 | 801 | 0.036079182 | 0.385566655 |
| GO:0050810 | regulation of steroid biosynthetic process | 5 | 32 | 0.03622843 | 0.385566655 |
| GO:0006144 | purine nucleobase metabolic process | 4 | 22 | 0.0362672 | 0.385566655 |
| GO:0035010 | encapsulation of foreign target | 4 | 22 | 0.0362672 | 0.385566655 |
| GO:0019438 | aromatic compound biosynthetic process | 149 | 2236 | 0.036277579 | 0.385566655 |
| GO:0008206 | bile acid metabolic process | 3 | 13 | 0.036440436 | 0.385566655 |
| GO:0031034 | myosin filament assembly | 3 | 13 | 0.036440436 | 0.385566655 |
| GO:0055070 | copper ion homeostasis | 3 | 13 | 0.036440436 | 0.385566655 |
| GO:1902993 | positive regulation of amyloid precursor protein catabolic process | 3 | 13 | 0.036440436 | 0.385566655 |
| GO:0018130 | heterocycle biosynthetic process | 148 | 2221 | 0.036904425 | 0.387179949 |
| GO:0001505 | regulation of neurotransmitter levels | 15 | 155 | 0.036964467 | 0.387179949 |
| GO:0006635 | fatty acid beta-oxidation | 6 | 43 | 0.037296638 | 0.387179949 |
| GO:0009201 | ribonucleoside triphosphate biosynthetic process | 6 | 43 | 0.037296638 | 0.387179949 |
| GO:0015918 | sterol transport | 6 | 43 | 0.037296638 | 0.387179949 |
| GO:0050832 | defense response to fungus | 6 | 43 | 0.037296638 | 0.387179949 |
| GO:0042439 | ethanolamine-containing compound metabolic process | 8 | 66 | 0.037318549 | 0.387179949 |
| GO:0065007 | biological regulation | 380 | 6123 | 0.037805016 | 0.391140544 |
| GO:1901362 | organic cyclic compound biosynthetic process | 155 | 2338 | 0.03820754 | 0.394213157 |
| GO:0042330 | taxis | 34 | 426 | 0.038735237 | 0.398556776 |
| GO:0090304 | nucleic acid metabolic process | 224 | 3482 | 0.039810149 | 0.408491505 |
| GO:0042246 | tissue regeneration | 5 | 33 | 0.040724844 | 0.416732308 |
| GO:0016311 | dephosphorylation | 18 | 198 | 0.040877669 | 0.417153257 |
| GO:0046532 | regulation of photoreceptor cell differentiation | 6 | 44 | 0.041141636 | 0.418703022 |
| GO:0042127 | regulation of cell proliferation | 41 | 533 | 0.041555976 | 0.421770575 |
| GO:0009147 | pyrimidine nucleoside triphosphate metabolic process | 4 | 23 | 0.041943672 | 0.423404363 |
| GO:0071711 | basement membrane organization | 4 | 23 | 0.041943672 | 0.423404363 |
| GO:0007416 | synapse assembly | 15 | 158 | 0.042648747 | 0.424327734 |
| GO:0002757 | immune response-activating signal transduction | 9 | 80 | 0.042728317 | 0.424327734 |
| GO:0008291 | acetylcholine metabolic process | 2 | 6 | 0.043566671 | 0.424327734 |
| GO:0009396 | folic acid-containing compound biosynthetic process | 2 | 6 | 0.043566671 | 0.424327734 |
| GO:0021535 | cell migration in hindbrain | 2 | 6 | 0.043566671 | 0.424327734 |
| GO:0060913 | cardiac cell fate determination | 2 | 6 | 0.043566671 | 0.424327734 |
| GO:0071624 | positive regulation of granulocyte chemotaxis | 2 | 6 | 0.043566671 | 0.424327734 |
| GO:0090022 | regulation of neutrophil chemotaxis | 2 | 6 | 0.043566671 | 0.424327734 |
| GO:0090023 | positive regulation of neutrophil chemotaxis | 2 | 6 | 0.043566671 | 0.424327734 |
| GO:0097049 | motor neuron apoptotic process | 2 | 6 | 0.043566671 | 0.424327734 |
| GO:1900619 | acetate ester metabolic process | 2 | 6 | 0.043566671 | 0.424327734 |
| GO:1902624 | positive regulation of neutrophil migration | 2 | 6 | 0.043566671 | 0.424327734 |
| GO:0001659 | temperature homeostasis | 3 | 14 | 0.044420922 | 0.424327734 |
| GO:0010742 | macrophage derived foam cell differentiation | 3 | 14 | 0.044420922 | 0.424327734 |
| GO:0015804 | neutral amino acid transport | 3 | 14 | 0.044420922 | 0.424327734 |
| GO:0034375 | high-density lipoprotein particle remodeling | 3 | 14 | 0.044420922 | 0.424327734 |
| GO:0034433 | steroid esterification | 3 | 14 | 0.044420922 | 0.424327734 |
| GO:0034434 | sterol esterification | 3 | 14 | 0.044420922 | 0.424327734 |
| GO:0034435 | cholesterol esterification | 3 | 14 | 0.044420922 | 0.424327734 |
| GO:0060415 | muscle tissue morphogenesis | 3 | 14 | 0.044420922 | 0.424327734 |
| GO:0090077 | foam cell differentiation | 3 | 14 | 0.044420922 | 0.424327734 |
| GO:0007189 | adenylate cyclase-activating G-protein coupled receptor signaling pathway | 6 | 45 | 0.045227822 | 0.430933461 |
| GO:0046890 | regulation of lipid biosynthetic process | 8 | 69 | 0.046752392 | 0.444326171 |
| GO:0008037 | cell recognition | 16 | 174 | 0.047483644 | 0.450130485 |
| GO:0009260 | ribonucleotide biosynthetic process | 14 | 147 | 0.047945501 | 0.452455392 |
| GO:0006536 | glutamate metabolic process | 4 | 24 | 0.048092313 | 0.452455392 |
| GO:0008345 | larval locomotory behavior | 4 | 24 | 0.048092313 | 0.452455392 |
| GO:0002764 | immune response-regulating signaling pathway | 9 | 82 | 0.048801053 | 0.457969684 |
| GO:0009620 | response to fungus | 6 | 46 | 0.049557968 | 0.461375708 |

# Table S4 GO enrichment of contracted genes in *L.decemlineata* (p <0.05, FDR adjust p as p.adjust)

| GO ID | Description | out | All | pvalue | p.adjust |
| --- | --- | --- | --- | --- | --- |
| GO:0005549 | odorant binding | 31 | 80 | 2.40E-24 | 1.57E-21 |
| GO:0030246 | carbohydrate binding | 40 | 149 | 4.19E-24 | 1.57E-21 |
| GO:0004984 | olfactory receptor activity | 24 | 60 | 1.67E-19 | 4.18E-17 |
| GO:0015114 | phosphate ion transmembrane transporter activity | 16 | 32 | 2.52E-15 | 4.73E-13 |
| GO:0004888 | transmembrane signaling receptor activity | 53 | 435 | 5.66E-15 | 8.50E-13 |
| GO:0099600 | transmembrane receptor activity | 56 | 486 | 1.04E-14 | 1.30E-12 |
| GO:0038023 | signaling receptor activity | 56 | 504 | 4.98E-14 | 5.34E-12 |
| GO:0048029 | monosaccharide binding | 16 | 38 | 7.63E-14 | 7.16E-12 |
| GO:0004872 | receptor activity | 61 | 612 | 4.48E-13 | 3.36E-11 |
| GO:0060089 | molecular transducer activity | 61 | 612 | 4.48E-13 | 3.36E-11 |
| GO:0005537 | mannose binding | 10 | 14 | 3.21E-12 | 2.19E-10 |
| GO:0004871 | signal transducer activity | 56 | 577 | 1.32E-11 | 8.26E-10 |
| GO:0033691 | sialic acid binding | 9 | 12 | 2.05E-11 | 1.18E-09 |
| GO:0047886 | farnesol dehydrogenase activity | 8 | 9 | 2.50E-11 | 1.25E-09 |
| GO:0070330 | aromatase activity | 8 | 9 | 2.50E-11 | 1.25E-09 |
| GO:0004190 | aspartic-type endopeptidase activity | 20 | 95 | 1.47E-10 | 6.50E-09 |
| GO:0070001 | aspartic-type peptidase activity | 20 | 95 | 1.47E-10 | 6.50E-09 |
| GO:0080019 | fatty-acyl-CoA reductase (alcohol-forming) activity | 11 | 26 | 6.13E-10 | 2.55E-08 |
| GO:0015103 | inorganic anion transmembrane transporter activity | 18 | 85 | 1.12E-09 | 4.42E-08 |
| GO:0070492 | oligosaccharide binding | 8 | 12 | 1.25E-09 | 4.69E-08 |
| GO:0045735 | nutrient reservoir activity | 6 | 6 | 2.24E-09 | 7.98E-08 |
| GO:0016712 | oxidoreductase activity, acting on paired donors, with incorporation or reduction of molecular oxygen, reduced flavin or flavoprotein as one donor, and incorporation of one atom of oxygen | 8 | 13 | 3.15E-09 | 1.07E-07 |
| GO:0015291 | secondary active transmembrane transporter activity | 26 | 186 | 3.41E-09 | 1.11E-07 |
| GO:0015925 | galactosidase activity | 8 | 14 | 7.11E-09 | 2.22E-07 |
| GO:0005506 | iron ion binding | 24 | 168 | 8.93E-09 | 2.68E-07 |
| GO:0020037 | heme binding | 25 | 184 | 1.24E-08 | 3.56E-07 |
| GO:0015293 | symporter activity | 21 | 134 | 1.46E-08 | 4.04E-07 |
| GO:0046906 | tetrapyrrole binding | 26 | 200 | 1.60E-08 | 4.30E-07 |
| GO:0004565 | beta-galactosidase activity | 7 | 11 | 2.33E-08 | 6.03E-07 |
| GO:0016491 | oxidoreductase activity | 62 | 878 | 2.91E-07 | 7.27E-06 |
| GO:0043274 | phospholipase binding | 6 | 10 | 4.14E-07 | 1.00E-05 |
| GO:0004497 | monooxygenase activity | 20 | 149 | 4.40E-07 | 1.03E-05 |
| GO:0022892 | substrate-specific transporter activity | 62 | 897 | 6.10E-07 | 1.39E-05 |
| GO:0015075 | ion transmembrane transporter activity | 50 | 666 | 7.89E-07 | 1.74E-05 |
| GO:0008509 | anion transmembrane transporter activity | 27 | 259 | 8.35E-07 | 1.79E-05 |
| GO:0022804 | active transmembrane transporter activity | 34 | 383 | 1.39E-06 | 2.90E-05 |
| GO:0038024 | cargo receptor activity | 12 | 67 | 4.49E-06 | 9.10E-05 |
| GO:0016903 | oxidoreductase activity, acting on the aldehyde or oxo group of donors | 13 | 80 | 5.57E-06 | 0.000108 |
| GO:0016705 | oxidoreductase activity, acting on paired donors, with incorporation or reduction of molecular oxygen | 20 | 175 | 5.64E-06 | 0.000108 |
| GO:0022891 | substrate-specific transmembrane transporter activity | 55 | 816 | 6.01E-06 | 0.000113 |
| GO:0015277 | kainate selective glutamate receptor activity | 5 | 10 | 1.35E-05 | 0.000246 |
| GO:0005215 | transporter activity | 65 | 1052 | 1.42E-05 | 0.000253 |
| GO:0051959 | dynein light intermediate chain binding | 8 | 34 | 2.26E-05 | 0.000395 |
| GO:0022857 | transmembrane transporter activity | 60 | 964 | 2.55E-05 | 0.000434 |
| GO:0045502 | dynein binding | 9 | 47 | 4.08E-05 | 0.00068 |
| GO:0016620 | oxidoreductase activity, acting on the aldehyde or oxo group of donors, NAD or NADP as acceptor | 11 | 71 | 4.63E-05 | 0.000755 |
| GO:0005509 | calcium ion binding | 27 | 322 | 4.73E-05 | 0.000755 |
| GO:0008569 | ATP-dependent microtubule motor activity, minus-end-directed | 8 | 38 | 5.36E-05 | 0.000838 |
| GO:0004970 | ionotropic glutamate receptor activity | 6 | 20 | 5.60E-05 | 0.000858 |
| GO:0045505 | dynein intermediate chain binding | 8 | 39 | 6.53E-05 | 0.00098 |
| GO:0099507 | ligand-gated ion channel activity involved in regulation of presynaptic membrane potential | 4 | 8 | 0.000107 | 0.001529 |
| GO:0031406 | carboxylic acid binding | 13 | 105 | 0.000108 | 0.001529 |
| GO:0043177 | organic acid binding | 13 | 105 | 0.000108 | 0.001529 |
| GO:0004453 | juvenile-hormone esterase activity | 5 | 16 | 0.000195 | 0.002653 |
| GO:1904315 | transmitter-gated ion channel activity involved in regulation of postsynaptic membrane potential | 5 | 16 | 0.000195 | 0.002653 |
| GO:0008066 | glutamate receptor activity | 6 | 25 | 0.000219 | 0.002928 |
| GO:0005230 | extracellular ligand-gated ion channel activity | 7 | 35 | 0.000223 | 0.002928 |
| GO:0099529 | neurotransmitter receptor activity involved in regulation of postsynaptic membrane potential | 5 | 18 | 0.000359 | 0.004646 |
| GO:0005534 | galactose binding | 3 | 5 | 0.000452 | 0.005744 |
| GO:0016616 | oxidoreductase activity, acting on the CH-OH group of donors, NAD or NADP as acceptor | 18 | 212 | 0.000736 | 0.009197 |
| GO:0016788 | hydrolase activity, acting on ester bonds | 55 | 979 | 0.000764 | 0.009387 |
| GO:0003777 | microtubule motor activity | 9 | 70 | 0.00093 | 0.011247 |
| GO:0005234 | extracellular-glutamate-gated ion channel activity | 4 | 13 | 0.000949 | 0.011294 |
| GO:0004312 | fatty acid synthase activity | 5 | 22 | 0.000979 | 0.011474 |
| GO:0004175 | endopeptidase activity | 27 | 392 | 0.001114 | 0.01285 |
| GO:0001965 | G-protein alpha-subunit binding | 4 | 14 | 0.00129 | 0.013549 |
| GO:0004031 | aldehyde oxidase activity | 2 | 2 | 0.001319 | 0.013549 |
| GO:0004772 | sterol O-acyltransferase activity | 2 | 2 | 0.001319 | 0.013549 |
| GO:0016623 | oxidoreductase activity, acting on the aldehyde or oxo group of donors, oxygen as acceptor | 2 | 2 | 0.001319 | 0.013549 |
| GO:0018488 | aryl-aldehyde oxidase activity | 2 | 2 | 0.001319 | 0.013549 |
| GO:0034736 | cholesterol O-acyltransferase activity | 2 | 2 | 0.001319 | 0.013549 |
| GO:0042806 | fucose binding | 2 | 2 | 0.001319 | 0.013549 |
| GO:0050302 | indole-3-acetaldehyde oxidase activity | 2 | 2 | 0.001319 | 0.013549 |
| GO:0015276 | ligand-gated ion channel activity | 12 | 124 | 0.001841 | 0.018411 |
| GO:0022834 | ligand-gated channel activity | 12 | 124 | 0.001841 | 0.018411 |
| GO:0022824 | transmitter-gated ion channel activity | 6 | 37 | 0.001991 | 0.019396 |
| GO:0022835 | transmitter-gated channel activity | 6 | 37 | 0.001991 | 0.019396 |
| GO:0001618 | virus receptor activity | 4 | 16 | 0.002214 | 0.021291 |
| GO:0004185 | serine-type carboxypeptidase activity | 3 | 8 | 0.002331 | 0.021826 |
| GO:0070008 | serine-type exopeptidase activity | 3 | 8 | 0.002331 | 0.021826 |
| GO:0046914 | transition metal ion binding | 54 | 1006 | 0.002357 | 0.021826 |
| GO:0043169 | cation binding | 160 | 3646 | 0.002605 | 0.023681 |
| GO:0052689 | carboxylic ester hydrolase activity | 16 | 200 | 0.002621 | 0.023681 |
| GO:0046872 | metal ion binding | 159 | 3622 | 0.002672 | 0.023854 |
| GO:0080030 | methyl indole-3-acetate esterase activity | 7 | 53 | 0.002906 | 0.025644 |
| GO:0016614 | oxidoreductase activity, acting on CH-OH group of donors | 19 | 264 | 0.003627 | 0.031627 |
| GO:0004555 | alpha,alpha-trehalase activity | 2 | 3 | 0.003861 | 0.03182 |
| GO:0005344 | oxygen transporter activity | 2 | 3 | 0.003861 | 0.03182 |
| GO:0015927 | trehalase activity | 2 | 3 | 0.003861 | 0.03182 |
| GO:0052894 | norspermine:oxygen oxidoreductase activity | 2 | 3 | 0.003861 | 0.03182 |
| GO:0052895 | N1-acetylspermine:oxygen oxidoreductase (N1-acetylspermidine-forming) activity | 2 | 3 | 0.003861 | 0.03182 |
| GO:0008233 | peptidase activity | 34 | 580 | 0.00409 | 0.033339 |
| GO:0015297 | antiporter activity | 6 | 44 | 0.004883 | 0.039379 |
| GO:0016787 | hydrolase activity | 123 | 2744 | 0.005018 | 0.040037 |
| GO:0005231 | excitatory extracellular ligand-gated ion channel activity | 4 | 20 | 0.005254 | 0.041478 |
| GO:0004553 | hydrolase activity, hydrolyzing O-glycosyl compounds | 13 | 159 | 0.005316 | 0.041529 |
| GO:0003774 | motor activity | 10 | 107 | 0.005515 | 0.04264 |
| GO:0070011 | peptidase activity, acting on L-amino acid peptides | 33 | 572 | 0.005888 | 0.045062 |
| GO:0015136 | sialic acid transmembrane transporter activity | 3 | 11 | 0.006332 | 0.047969 |
| GO:0015267 | channel activity | 19 | 279 | 0.006537 | 0.049024 |
| GO:0003887 | DNA-directed DNA polymerase activity | 6 | 47 | 0.00678 | 0.049882 |
| GO:0022803 | passive transmembrane transporter activity | 19 | 280 | 0.006784 | 0.049882 |
| GO:0005216 | ion channel activity | 18 | 262 | 0.00733 | 0.052821 |
| GO:0016647 | oxidoreductase activity, acting on the CH-NH group of donors, oxygen as acceptor | 2 | 4 | 0.007536 | 0.052821 |
| GO:0046592 | polyamine oxidase activity | 2 | 4 | 0.007536 | 0.052821 |
| GO:0047238 | glucuronosyl-N-acetylgalactosaminyl-proteoglycan 4-beta-N-acetylgalactosaminyltransferase activity | 2 | 4 | 0.007536 | 0.052821 |
| GO:0052901 | spermine:oxygen oxidoreductase (spermidine-forming) activity | 2 | 4 | 0.007536 | 0.052821 |
| GO:0022838 | substrate-specific channel activity | 18 | 263 | 0.007613 | 0.052866 |
| GO:0015174 | basic amino acid transmembrane transporter activity | 3 | 12 | 0.008217 | 0.05654 |
| GO:0030594 | neurotransmitter receptor activity | 7 | 64 | 0.008331 | 0.056805 |
| GO:0022836 | gated channel activity | 15 | 207 | 0.008658 | 0.058502 |
| GO:0004315 | 3-oxoacyl-[acyl-carrier-protein] synthase activity | 3 | 13 | 0.010398 | 0.069628 |
| GO:0000064 | L-ornithine transmembrane transporter activity | 2 | 5 | 0.012258 | 0.079257 |
| GO:0019825 | oxygen binding | 2 | 5 | 0.012258 | 0.079257 |
| GO:0048039 | ubiquinone binding | 2 | 5 | 0.012258 | 0.079257 |
| GO:0061459 | L-arginine transmembrane transporter activity | 2 | 5 | 0.012258 | 0.079257 |
| GO:0016798 | hydrolase activity, acting on glycosyl bonds | 14 | 200 | 0.014542 | 0.093216 |
| GO:0030159 | receptor signaling complex scaffold activity | 3 | 15 | 0.015675 | 0.099627 |
| GO:0008093 | cytoskeletal adaptor activity | 2 | 6 | 0.017948 | 0.111248 |
| GO:0009922 | fatty acid elongase activity | 2 | 6 | 0.017948 | 0.111248 |
| GO:0015189 | L-lysine transmembrane transporter activity | 2 | 6 | 0.017948 | 0.111248 |
| GO:1901681 | sulfur compound binding | 8 | 94 | 0.020925 | 0.128638 |
| GO:0032947 | protein complex scaffold | 3 | 17 | 0.022203 | 0.135174 |
| GO:0003993 | acid phosphatase activity | 4 | 30 | 0.022349 | 0.135174 |
| GO:0004143 | diacylglycerol kinase activity | 2 | 7 | 0.024528 | 0.142606 |
| GO:0015181 | arginine transmembrane transporter activity | 2 | 7 | 0.024528 | 0.142606 |
| GO:0016635 | oxidoreductase activity, acting on the CH-CH group of donors, quinone or related compound as acceptor | 2 | 7 | 0.024528 | 0.142606 |
| GO:0043295 | glutathione binding | 2 | 7 | 0.024528 | 0.142606 |
| GO:1900750 | oligopeptide binding | 2 | 7 | 0.024528 | 0.142606 |
| GO:0015179 | L-amino acid transmembrane transporter activity | 4 | 31 | 0.024944 | 0.14391 |
| GO:0072341 | modified amino acid binding | 5 | 49 | 0.031893 | 0.175904 |
| GO:0004180 | carboxypeptidase activity | 4 | 34 | 0.033784 | 0.175904 |
| GO:0003964 | RNA-directed DNA polymerase activity | 13 | 204 | 0.035507 | 0.175904 |
| GO:0002054 | nucleobase binding | 1 | 1 | 0.036353 | 0.175904 |
| GO:0002058 | uracil binding | 1 | 1 | 0.036353 | 0.175904 |
| GO:0002059 | thymine binding | 1 | 1 | 0.036353 | 0.175904 |
| GO:0002061 | pyrimidine nucleobase binding | 1 | 1 | 0.036353 | 0.175904 |
| GO:0003979 | UDP-glucose 6-dehydrogenase activity | 1 | 1 | 0.036353 | 0.175904 |
| GO:0004082 | bisphosphoglycerate mutase activity | 1 | 1 | 0.036353 | 0.175904 |
| GO:0004157 | dihydropyrimidinase activity | 1 | 1 | 0.036353 | 0.175904 |
| GO:0004351 | glutamate decarboxylase activity | 1 | 1 | 0.036353 | 0.175904 |
| GO:0004556 | alpha-amylase activity | 1 | 1 | 0.036353 | 0.175904 |
| GO:0004656 | procollagen-proline 4-dioxygenase activity | 1 | 1 | 0.036353 | 0.175904 |
| GO:0004820 | glycine-tRNA ligase activity | 1 | 1 | 0.036353 | 0.175904 |
| GO:0004850 | uridine phosphorylase activity | 1 | 1 | 0.036353 | 0.175904 |
| GO:0005219 | ryanodine-sensitive calcium-release channel activity | 1 | 1 | 0.036353 | 0.175904 |
| GO:0005290 | L-histidine transmembrane transporter activity | 1 | 1 | 0.036353 | 0.175904 |
| GO:0005476 | carnitine:acyl carnitine antiporter activity | 1 | 1 | 0.036353 | 0.175904 |
| GO:0016160 | amylase activity | 1 | 1 | 0.036353 | 0.175904 |
| GO:0019798 | procollagen-proline dioxygenase activity | 1 | 1 | 0.036353 | 0.175904 |
| GO:0031492 | nucleosomal DNA binding | 1 | 1 | 0.036353 | 0.175904 |
| GO:0043237 | laminin-1 binding | 1 | 1 | 0.036353 | 0.175904 |
| GO:0047237 | glucuronylgalactosylproteoglycan 4-beta-N-acetylgalactosaminyltransferase activity | 1 | 1 | 0.036353 | 0.175904 |
| GO:0060072 | large conductance calcium-activated potassium channel activity | 1 | 1 | 0.036353 | 0.175904 |
| GO:0099580 | ion antiporter activity involved in regulation of postsynaptic membrane potential | 1 | 1 | 0.036353 | 0.175904 |
| GO:0005221 | intracellular cyclic nucleotide activated cation channel activity | 3 | 21 | 0.039034 | 0.185288 |
| GO:0005222 | intracellular cAMP activated cation channel activity | 3 | 21 | 0.039034 | 0.185288 |
| GO:0043855 | cyclic nucleotide-gated ion channel activity | 3 | 21 | 0.039034 | 0.185288 |
| GO:0015368 | calcium:cation antiporter activity | 2 | 9 | 0.040077 | 0.189042 |
| GO:0030674 | protein binding, bridging | 5 | 53 | 0.042723 | 0.200263 |
| GO:0030165 | PDZ domain binding | 5 | 55 | 0.048873 | 0.22645 |
| GO:0043208 | glycosphingolipid binding | 2 | 10 | 0.048913 | 0.22645 |
